# A Computational–Experimental Investigation of the Molecular Mechanism of Interleukin-6-Piperine Interaction

**DOI:** 10.3390/ijms23147994

**Published:** 2022-07-20

**Authors:** Ana Paula Ribeiro Povinelli, Gabriel Zazeri, Alan M. Jones, Marinônio Lopes Cornélio

**Affiliations:** 1 Federal Institute of Education, Science and Technology of Mato Grosso, Campo Novo do Parecis 78360-000, Brazil; ana.povinelli@colaborador.ifmt.edu.br; 2 School of Pharmacy, Institute of Clinical Sciences, College of Medical and Dental Sciences, University of Birmingham, Edgbaston, Birmingham B15 2TT, UK; a.m.jones.2@bham.ac.uk; 3 Departamento de Física, Instituto de Biociências, Letras e Ciências Exatas (IBILCE), UNESP, Rua Cristovão Colombo 2265, São José do Rio Preto 15054-000, Brazil

**Keywords:** piperine, Interleukin-6, IL6, fluorescence spectroscopy, molecular docking, umbrella sampling, molecular biophysics

## Abstract

Herein, we elucidate the biophysical aspects of the interaction of an important protein, Interleukin-6 (IL6), which is involved in cytokine storm syndrome, with a natural product with anti-inflammatory activity, piperine. Despite the role of piperine in the inhibition of the transcriptional protein NF-κB pathway responsible for activation of IL6 gene expression, there are no studies to the best of our knowledge regarding the characterisation of the molecular interaction of the IL6-piperine complex. In this context, the characterisation was performed with spectroscopic experiments aided by molecular modelling. Fluorescence spectroscopy alongside van’t Hoff analyses showed that the complexation event is a spontaneous process driven by non-specific interactions. Circular dichroism aided by molecular dynamics revealed that piperine caused local α-helix reduction. Molecular docking and molecular dynamics disclosed the microenvironment of interaction as non-polar amino acid residues. Although piperine has three available hydrogen bond acceptors, only one hydrogen-bond was formed during our simulation experiments, reinforcing the major role of non-specific interactions that we observed experimentally. Root mean square deviation (RMSD) and hydrodynamic radii revealed that the IL6-piperine complex was stable during 800 ns of simulation. Taken together, these results can support ongoing IL6 drug discovery efforts.

## 1. Introduction

Human interleukin-6 (IL6) is a 26 kDa protein that was primarily identified as a regulator of B-cell differentiation [1,2]. IL6 is expressed by several types of cells, including monocytes, macrophages, lymphocytes, fibroblasts, keratinocytes, endothelial cells, and certain tumour cells [3]. IL6 is a pleiotropic cytokine with significant functions in the regulation of the immune system [4]. IL6 gene expression is activated by the transcriptional protein NF-κB, and once expressed, IL6 exhibits a potent pro-inflammatory activity [5]. However, increased or deregulated expression of IL-6 substantially contributes to the pathogenesis of chronic inflammatory diseases [2], and it is also associated with acute diseases (e.g., COVID-19, in which the cytokine storm is triggered during SARS-CoV-2 infection [6,7]).

The IL6 structure is composed of 185 amino acids, with one tryptophan residue at position 158 (W158). Tryptophan has a high quantum yield, which makes fluorescence spectroscopy an appropriate methodology for the study of IL6. IL6 has four cysteine amino acids that form two disulfide bonds, conferring some chemical and thermal resistance to the protein native structure [8,9]. IL6 3D structure is composed of α-helix contents rearranged as five α-helices interspersed with different length loops; four of these helices constitute a classical four-helix bundle linked by loops, and the final helix is a mini-helix [10,11].

Many efforts have been made to target IL6. Most of the drugs that have been studied are monoclonal antibodies (e.g., clazakizumab, olokizumab, sirukumab, siltuximab and ziltivekimab) [12]. The possibility of targeting IL6 with small molecules has been investigated recently [13].

**Figure 1:**
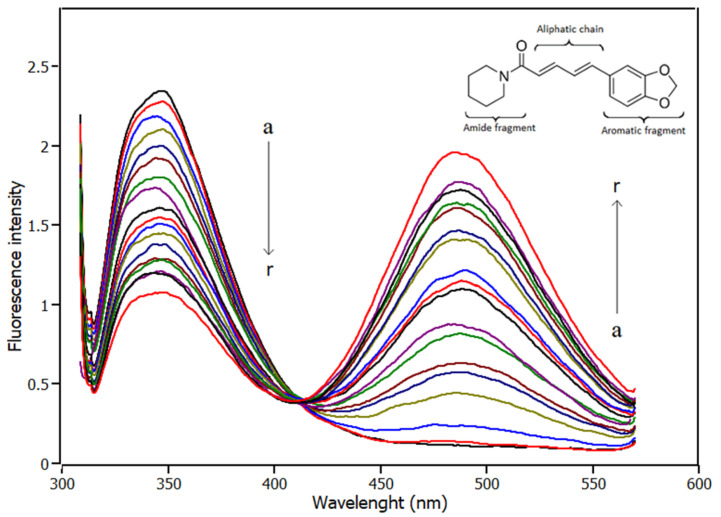
Spectra of fluorescence emission of IL6 obtained from titration experiments with increments in the concentration of piperine (pH 7.4, T = 288 K, λ_excitation_ = 295 nm). [IL6] = 4.0 μM; piperine titrations with increment of 1.0 μM in different colors (a → r 0.0 μM → 17.0 μM).

In this work, we show that piperine, an alkaloid amide found in some *piper* species [14], interacts with IL6. [15] Piperine inhibits the NF-κB inflammation pathway, leading to the downregulation of pro-inflammatory proteins in human and rats [16,17,18]. The molecular structure of piperine (Figure 1) is composed of two groups, electronically conjugated-benzodioxole and pentadienone chromophore [19]. This structural feature leads to characteristic ultraviolet (UV) absorptions [20]. A review reported in the literature this year (2022) gathered a set of studies of important targets that interact (experimental and computational results) with piperine [21]. This set of proteins is composed by inflammatory cytokines (IL-8, IL-10, IL-1 h, IL-6), transcriptional factors (AP-1, NF-κB, ATF-2), kinases (JNK, ERK1, ERK2, Akt, P38 MAPK), enzymes (NOS-2, COX-2, MMP-2, MMP-9), transporter genes (CYP3A4, RV1258c, MRP1, BCRP), and cell-cycle proteins (cyclin A/D/T, CDK-2, CDK-4), among others.

Although piperine has exhibited biological activity on the inhibition of the inflammatory NF-κB pathway in cellular and in vivo experiments, the molecular targets and interactions have not been fully disclosed. In our previous studies, we elucidated the mode of binding and affinity of piperine toward some important targets of the NF-κB inflammation pathway, such as Interleukin-1β [20], nucleotide-binding domain of heat shock protein 70 [22] and nuclear factor kappa B (NF-κB) [15]. In order to move forward in our investigations, in this work we present the spectroscopic and computational biophysical characterisation of the IL6-piperine complex to elucidate the main features of the interaction and small-molecule drug discovery efforts in this emerging topic.

## 2. Results and Discussion

### 2.1. Fluorescence Spectroscopy

Figure 1 shows the results in the IL6 Trp158 fluorescence quenching caused by the addition of piperine to the solution. Interpreting the spectra, there are two fluorescent bands centred at 340 nm and 485 nm, which refer to Trp158 fluorescence emission and to piperine fluorescence emission, respectively. The fluorescence intensity of tryptophan emission decreased while piperine was added to the solution, which evidenced that the Trp158 fluorescence was quenched. Another characteristic observed is that the Trp158 fluorescence band remained centred at 340 nm during piperine titration, which indicates the fluorophore was not exposed to an environment with different polarity [23]. Another feature observed in the emission fluorescence spectrum is that for the maximum concentration of piperine, the full width half maximum (FWHM) of the bands at 340 nm and 485 nm is ±28 nm and ±43 nm, respectively. Thus, the bands did not overlap, allowing the fluorescence data to be analysed accurately.

There are two potential quenching mechanisms in operation [24]. One is dynamic quenching, where the ligand deactivate the excited form of the protein fluorophore through collisions—a process strongly influenced by thermal variations. The second mechanism is static quenching, characterised by the formation of a protein–ligand complex [25]. The quenching mechanisms can be differentiated by analysing the Stern–Volmer constants (K_SV_) at different temperatures [26] (Equation (1)), which relates the intensities of Trp158 fluorescence in the absence of piperine (F_0_) and in the presence of piperine (F). The system is under static quenching if the K_SV_ decreases or remains unchanged with rising temperature. On the other hand, if, with the increase in temperature, the K_SV_ increases, it is evidence of collisional processes.

Another feature that can be analysed is the linearity of Stern–Volmer function, in which the deviation of the Stern–Volmer function from linearity indicates that collisional quenching is present and that both mechanisms may be present in the system under study [26,27].

(1) $$\frac{F_{0}}{F}=1+K_{SV}\cdot \left[piperine\right]$$

**Figure 2:**
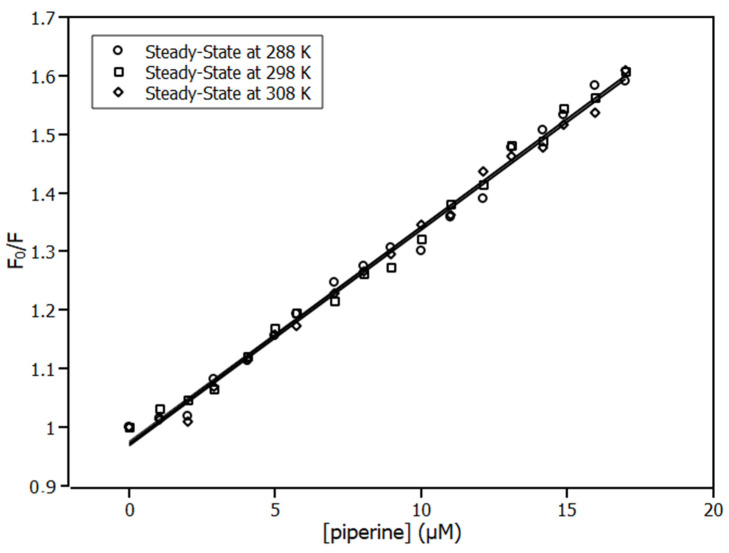
Stern–Volmer plots at three temperatures: 288 K, 298 K, and 308 K; [IL6] = 4.0 μM, [piperine] = 0.0–17.0 μM. R^2^ > 0.99.

The Stern–Volmer plots (Figure 2) exhibit linear response under piperine titration, showing that there is a single class of fluorophore in the protein, all equally accessible to the quencher, and therefore only one quenching mechanism occurred [27]–the static mechanism. According to the results, *K_SV_* remained unchanged as the temperature increased (Table 1). As discussed previously, these results revealed that the quenching mechanism was static; therefore, a complex was formed by IL6 and *piperine.*

**Figure 3:**
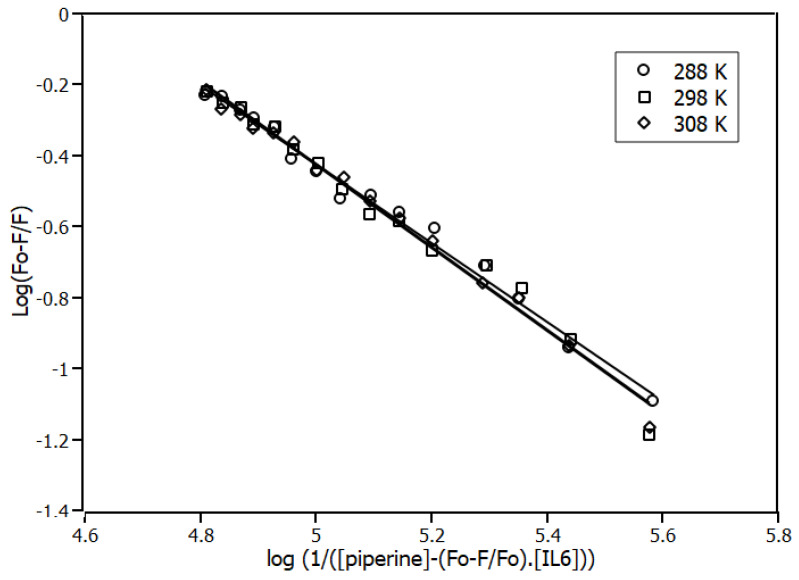
Double-log plots for the fluorescence quenching of IL6 (4.0 μM) in the presence of piperine at 288 K, 298 K and 308 K. R^2^ > 0.98.

In order to characterise the complex, the association constant, also known as binding constant (*K_a_*), was measured through the binding equilibrium model. The variable *K_a_* was obtained by linearising the function of the plot of Figure 3 using the double-logarithm equation (Equation (2)), which relates the quenching fluorescence intensities to the total concentration of piperine.

(2) $$\log\left(\frac{F_{0}-F}{F}\right)=n\cdot \log K_{a}-n\cdot \log\left(\frac{1}{\left[piperine\right]-\left(\frac{F_{0}-F}{F_{o}}\right)\cdot \left[IL6\right]}\right)$$

The results of *K_a_* at different temperatures obtained for the first order model (n ≈ 1) are shown in Table 1. The binding constants found for different temperatures were in the order magnitude of 10^4^ M^−1^. As shown in Table 1, the affinity of the complex was not influenced by temperature, since the results of the binding equilibrium experiments showed that *K_a_* was the same for the three temperatures. This behaviour reinforced the quenching mechanism. The affinity of piperine for IL6 was not influenced by an increase in the molecular motion of the system.

### 2.2. Thermodynamic Parameters

**Figure 4:**
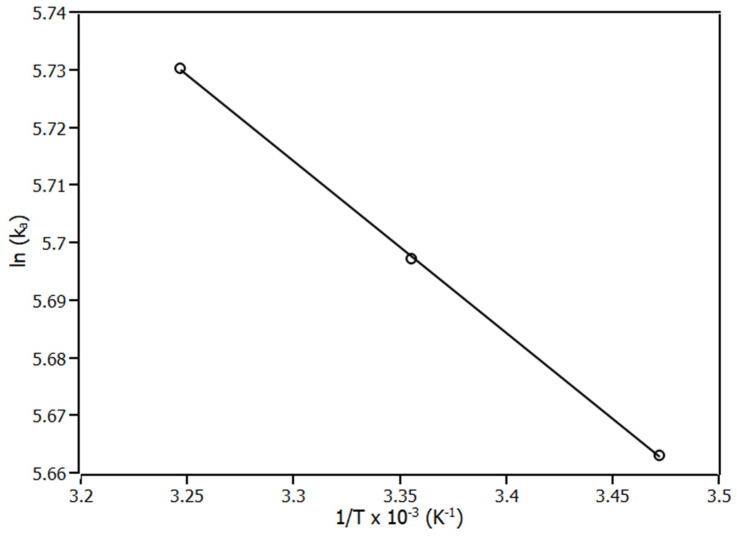
Van’t Hoff plot for the complex IL6-piperine at 288 K, 298 K, and 308 K. R^2^ > 0.99.

Based on thermodynamic parameters, such as ΔS (entropy variation), ΔH (enthalpy variation), and ΔG (Gibbs free variation) it was possible to acquire information about the spontaneity of complex formation, thermochemistry of complex formation and the forces that drove the complexation [28]. The parameters ΔS and ΔH can be obtained by linearisation of the van’t Hoff plot (Figure 4) by Equation (3), where ΔS is the linear coefficient and ΔH is the slope. With ΔS and ΔH, ΔG is obtained from Equation (4).

(3) $$\ln K_{a}=-\frac{\Delta H}{R.T}+\frac{\Delta S}{R}$$

(4) ΔG=ΔH−TΔS

The results of ΔS, ΔH, and ΔG are shown at Table 2. Regarding the results, ΔG values were negative for the three temperatures, showing that the complexation was a spontaneous process, independent of the system temperature. Furthermore, the values of ΔG have similar values for the three temperatures, showing that the increase in temperature did not influence the spontaneity of complexation.

Related to the thermochemistry of complexation, the process occurred with the absorption of heat (ΔH > 0), being an endothermic process. In addition, both terms T.ΔS and ΔH are positive values that reveal the non-specific interactions as the main interactions that drove the complexation [20,22,23]. This information is further reinforced in the molecular modelling section, which points to a single hydrogen bond formed in the complex.

### 2.3. Circular Dichroism Spectroscopy

**Figure 5:**
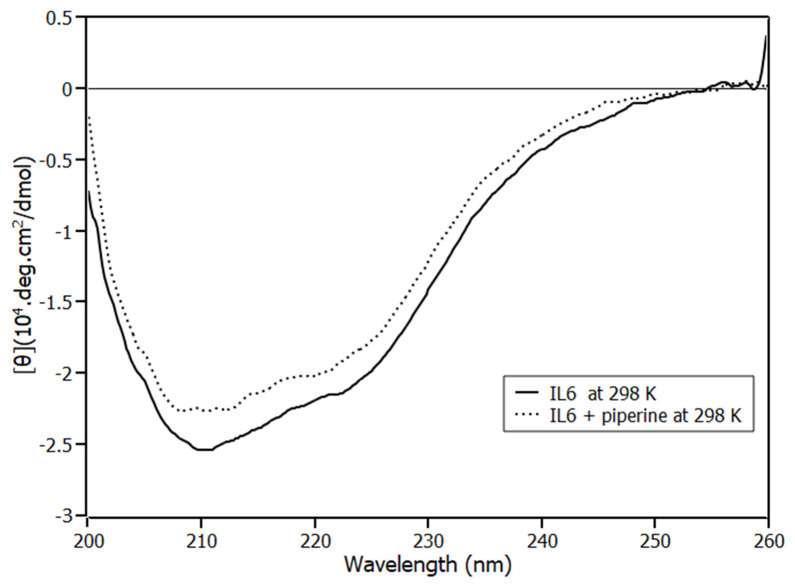
Circular dichroism of IL6 in the absence of piperine (solid lines) and IL6 with piperine at the stoichiometry 1:4 (dotted line) and at 298 K.

An investigation regarding protein-binding studies involves ascertaining whether the protein experiences conformational changes in the presence of the ligand. To elucidate this question, circular dichroism (CD) experiment was employed. The (CD) spectrum of IL6 (Figure 5) presented negative bands at 208 nm (π-π* transition) and 222 nm (n-π* transition), which are characteristics of alpha helix content [29], [30]. The same pattern of CD spectrum of IL6 in the absence of the ligand was obtained by Kruttgen, A. et al. [31] for human IL6, and by Zhang, J. et al. [32] for murine IL6.

In order to investigate the change in α helical structure percentage of IL6 on the addition of piperine, the CD spectra was analysed by applying Chen et al. [33] methodology (Equation (5)). In this methodology, we followed the mean residue ellipticity (*MRE*) at 222 nm.

(5) $$\%\;\alpha \;helix=\left(\frac{MRE_{222\;\mathrm{nm}}-2340}{30,300}\right)\cdot \;100$$

According to the analysis, IL6 had (63 ± 2)% α-*helices* in the absence of piperine. The result obtained for the protein in the absence of the ligand is in good agreement with the data reported in the literature for this same protein using CONTIN program (67% of α-helices) [31]. When the protein was in the presence of the *highest* concentration of piperine used in the binding equilibrium experiments (1:4), its structure had secondary fractions of (56 ± 2)% of alpha-*helices*, experiencing a reduction of 3% of α-*helix* content.

### 2.4. Molecular Modelling

**Figure 6:**
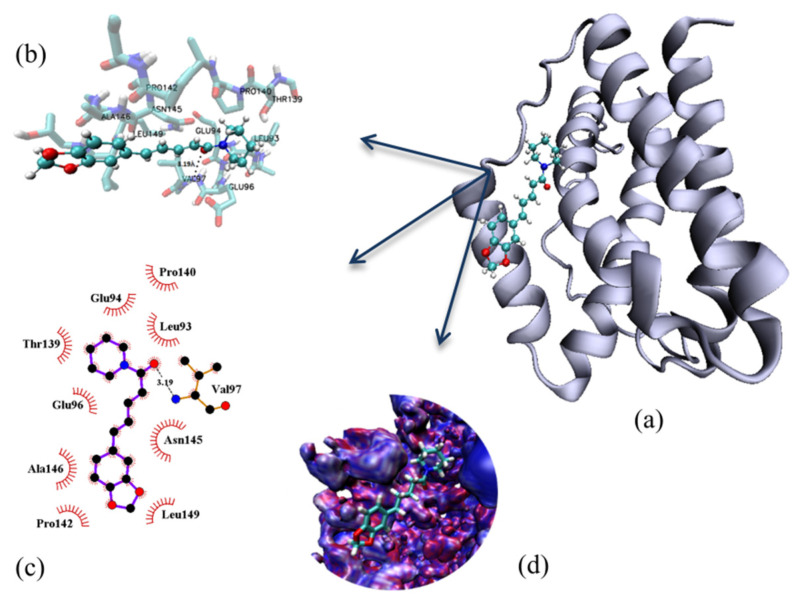
Microenvironment of interaction visualised with (**a**) pose of the complex obtained by molecular docking, (**b**) VMD (piperine is represented by ball and stick style and protein amino acids in licorice), (**c**) ligplot. In both visualisations, the hydrogen bond is stressed with dotted lines. (**d**) Theoretical electrostatic potential map (MEP) of IL6 and piperine at the binding site. MEP was obtained from APBS server, considering AMBER [34] as force field and pH 7.4.

Molecular docking was applied to predict the binding site found experimentally. The binding environments of the two sites are shown in Figure 6, and according to the results, the binding site is mainly composed by non-polar amino acids Pro140-142, Leu93-149, Ala145, Val97. The binding site is also composed of the polar amino acids Thr139 and Asn145, and the negatively charged amino acids Glu94-96. There is only one specific interaction (hydrogen bond) with a 3.19 Å distance formed by piperine and Val97. These results are in agreement with experimental van’t Hoff analysis, which indicated the non-specific interactions as the predominant interaction.

**Figure 7:**
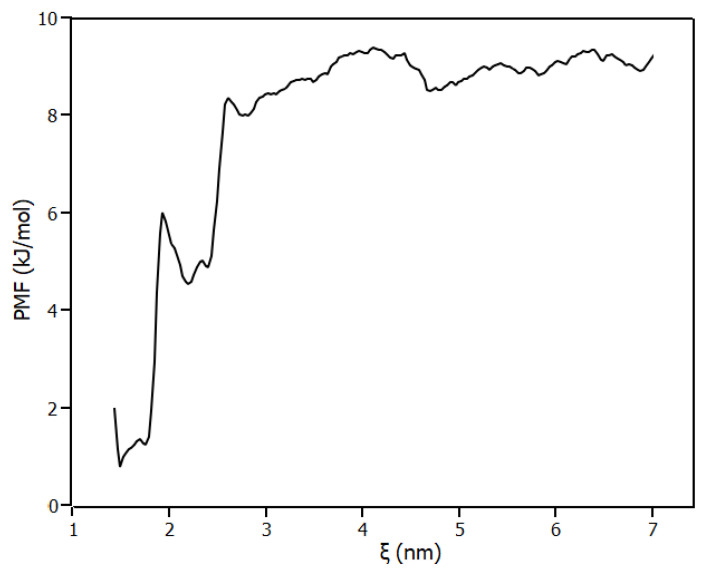
Potential of mean force (PMF) for the dissociation of piperine from IL6.

The most promising binding site predicted by molecular docking was confirmed by employing the umbrella sampling method calculated with molecular dynamics. Figure 7 shows the potential of mean force (PMF) of the complex dissociation. According to the result obtained, the PMF profile had the minimum of energy at the configuration predicted by molecular docking, which indicates that the pose configured a stable conformation. Moreover, the standard free energy for the binding site (ΔG_pred_) was determined by Weighted Histogram Analysis Method (WHAM), and regarding the results, the binding site had ΔG_pred_ = (−9 ± 2) kJ/mol similar to the ΔG found experimentally (ΔG = −14 ± 3) kJ/mol (Table 2). These results show that the experimental and computational methods were in agreement.

**Figure 8:**
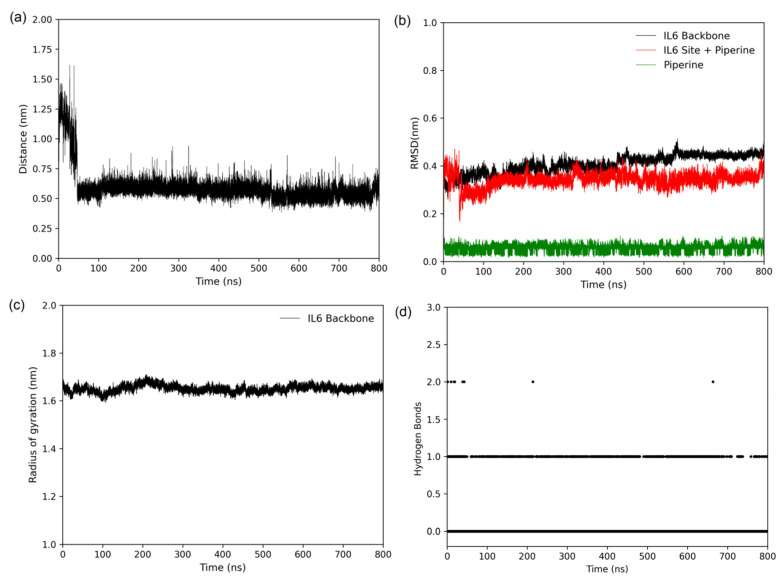
(**a**) Distance between the centres of geometry (COG) of IL6 and piperine (black). (**b**) Root mean square deviation (RMSD) calculated for the backbone of free IL6 (black), IL6 amino acids of binding site + piperine (red) and piperine (green). (**c**) Radius of gyration of IL6 with piperine in the binding site. (**d**) Number of hydrogen bonds formed between piperine and IL6 during the simulation.

The stability of the complex was verified during 800 ns of simulation. Figure 8a shows that the distance between the centre of geometry (COG) of piperine and the centre of geometry (COG) of the IL6 binding site decreased after 10 ns of simulation, revealing that piperine was buried into the accommodating binding site inside the pocket. From 10 ns to 800 ns, the distance of COGs remained at 0.6 nm. The temporal stability of the protein and piperine was verified by the root mean square deviation (RMSD) (Figure 8b) and radius of gyration (Rg) (Figure 8c). Figure 8b shows that the RMSDs of IL6 (in black), piperine (in green), and amino acids of the IL6 binding site + piperine (in red) remained stable during the 800 ns of simulation, with RMSD of about 0.4 nm, 0.07 nm, and 0.3 nm, respectively. Notably, that the variation in the RMSD profile of amino acids of IL6 + piperine at the beginning of simulation was due to the accommodation of piperine in the binding site revealed by the distance plot. The radius of gyration was another physical parameter followed to verify the stability of complex. According to Figure 8c, the radius remained at 1.65 nm during the simulation time frame, reinforcing the stability verified by RSMD analyses.

The stability of the specific interaction performed between IL6 and piperine—the hydrogen bond—was analysed during the simulation (Figure 8d). According to the analyses, piperine and IL6 formed either one or zero hydrogen bonds during the 800 ns of simulation, reinforcing the results obtained by van’t Hoff analyses and molecular docking, which revealed that the complexation was driven by non-specific interactions. Based on this result, we questioned whether the only hydrogen bond formed after the accommodation of piperine at the binding site remained between Val97 and piperine. Figure S4 shows all the hydrogen bonds formed during the simulation, and the amino acid residues that formed hydrogen bonds with piperine were Asn104, Arg105, and Gln153-all amino acids, which are polar (either charged or not). Despite the three amino acids being polar, and piperine having three hydrogen bond acceptors, the geometry of the interaction did not favour the formation of hydrogen bonds. An alternative to increase the affinity and specificity of interaction would be adding hydrogen bond donors to piperine analogues by synthetic modifications in future drug discovery studies.

**Figure 9:**
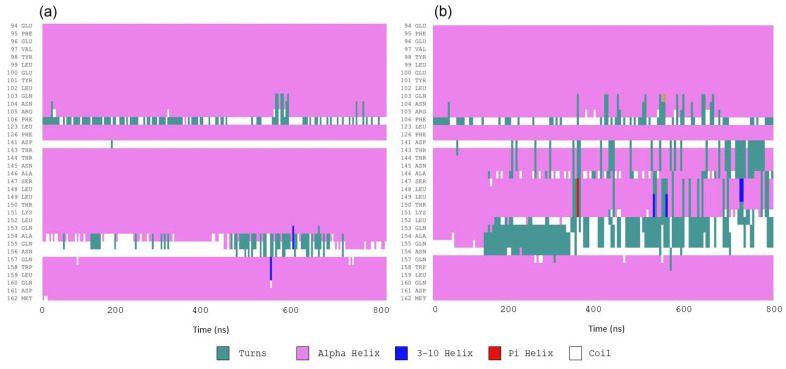
Local secondary structures of IL6 calculated during the molecular dynamics simulations in (**a**) the absence of piperine and (**b**) piperine inside the binding pocket.

**Figure 10:**
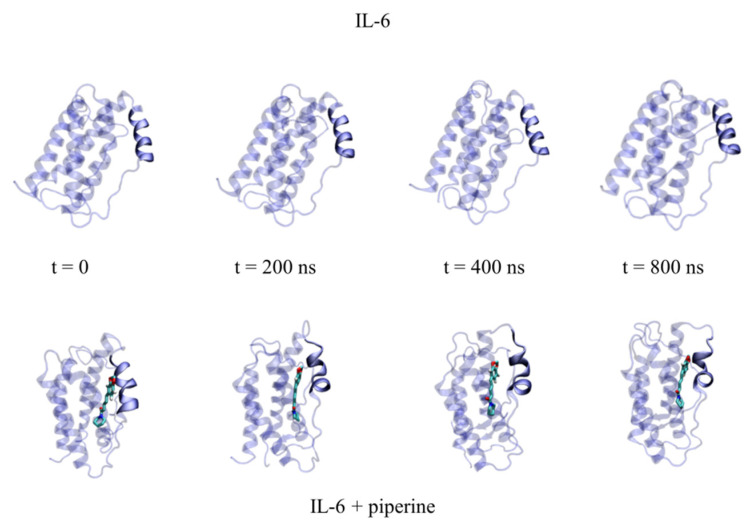
Temporal frames obtained from 0 ns to 800 ns of molecular dynamics simulation. Protein is represented by transparent new cartoon, with the opaque α-helix formed by the amino acids from Thr143 to Gln153. The top structures represent the protein in the absence of piperine. The bottom structures represent the complex protein–piperine throughout the simulation, with piperine represented by licorice.

According to circular dichroism results, IL6 experienced a reduction in α-*helix* content when it interacted with piperine. The secondary structures of the protein during 800 ns of simulation were used to verify in which regions of the protein the changes occurred. According to the results (Figure 9 and Figure S5), the reduction in α-*helix* occurred from the amino acids Thr143 to Gln153, in which the portions of α-helices were smoothly converted to coils and turns. The majority of these amino acid residues were present in the binding site, showing that piperine caused local conformational change (Figure 10).

## 3. Materials and Methods

### 3.1. Reagents

Piperine (>97%) was purchased from Sigma-Aldrich Chemical Co. (Schnelldorf, Bavaria, Germany), as dibasic sodium phosphate (>99%) reagents, anhydrous citric acid (>99%), and sodium chloride (>99%). Lyophilised IL6 (>97%) was purchased from GenScript. Methanol was purchased from Dynamics Química Contemporânea LTDA (Indaiatuba, SP, Brazil). All the materials purchased were used as supplied. Ultrapure water was prepared by a Millipore water purification system -Direct-Q UV-3 (Merck KGaA, Darmstadt, Germany). Lyophilised IL6 was reconstituted in 50 mM phosphate buffer containing 150 mM sodium chloride, and the pH was adjusted to 7.4 with anhydrous citric acid. Stock solutions of piperine were prepared in pure methanol. The concentrations of piperine and IL6 solutions were determined by UV-VIS experiments performed on Biospectro spectrophotometer (Biospectro, Curitiba, PR, Brazil), using the extinction coefficient at 16,500 M^−1^ cm^−1^ at 345 nm for piperine and 10,220 M^−1^ cm^−1^ at 280 nm for IL6.

### 3.2. Steady-State Fluorescence Spectroscopy

Fluorescence experiments were performed on the Lumina (Thermo Fisher Scientific, Waltham, MA, USA) stationary state spectrofluorimeter equipped with a thermal bath and Xenon lamp. A 100 μL quartz cuvette with a 10 × 2 mm optical path was used in the experiments. The widths of the excitation and emission slits were adjusted to 10 nm. The wavelength of 295 nm was used to excite the single tryptophan residue of IL6 (Trp158). The emission spectra were obtained in the range from 305 to 570 nm with a resolution of 1.0 ± 5.0 nm. Each emission point collected was the average of 15 accumulations. The software ScanWave was used to collect the measured data.

In the binding equilibrium experiments, aliquots of piperine (increment of 1 μM) were added into the IL6 solution at 4 μM. Measurements were performed at 288, 298, and 308 K. In all experiments, the final volume of methanol in the buffer was less than 1.0%.

The correction of the inner filter effects was achieved with Equation (6), where *F_corr_* and *F_obs_* are corrected and observed fluorescence intensities, and *A_ex_* and *A_em_* are the absorbance at the excitation and the emission wavelengths, respectively, considering a cuvette of 10 × 10 mm of optical path [26].

(6) $$F_{corr}=F_{obs}\cdot 10^{\frac{\left(5.A_{ex}+A_{em}\right)}{10}}$$

### 3.3. Circular Dichroism Spectroscopy

Circular dichroism spectra were recorded at 298 K on a Jasco J-815 spectropolarimeter model DRC-H (Jasco, Easton, MD, USA) equipped with a demountable quartz cell with a 0.01 cm optical path length. The CD spectra were recorded in the 200 to 260 nm range with a scan rate of 20 nm/min and a spectral resolution of 0.1 nm. For each spectrum, 15 accumulations were performed. The molar ratios of IL6 and piperine were 1:0 and 1:4, and the buffer spectrum was subtracted. The ellipticity θ collected in millidegrees was converted to mean residue ellipticity [θ] (deg cm^2^ dmol^−1^) using Equation (7). All the experiments were performed in triplicate.

(7) $$\left[\theta \right]=\frac{\theta \left(\mathrm{mdeg}\right)}{10\cdot \left[P\right]\cdot l\cdot n}$$

### 3.4. Molecular Docking

The piperine structures used in molecular docking were obtained from ab initio calculations from our previous work [29]. The AutoDockTools [35] software of the MGL program Tools 1.5.4 was used to prepare the IL6 (PDB 1 IL6) by adding polar hydrogen atoms and Gasteiger charges. The maps were generated by the AutoGrid 4.2 program [36] with a spacing of 0.375 Å, a dimension of 126 × 126 × 126 points, and grid centre coordinates of −0.201, 0.333, and 0.294 for x, y, and z coordinates, respectively. The AutoDock 4.2 program [35] was used to investigate the IL6 binding sites using the Lamarckian Genetic Algorithm (LGA) with a population size of 150, a maximum number of generations of 27,000, and energy evaluations equal to 2.5 × 10^6^. The other parameters were selected as software defaults. To generate different conformations, the total number of runs was set to 100. The final conformations were chosen among the most negative energies and belonging to the most representative cluster (Figure S1). The final conformations were visualised on VMD [37]. The binding microenvironment was generated by LigPlot [38].

### 3.5. Molecular Dynamics

The simulations of the complex IL6/piperine were performed with a GROMOS54a6 force field [39] by Gromacs v.5.1.4 [40]. The topology of piperine compatible with the force field was obtained from ATB webserver [41]. The complex was placed in a rectangular box, solvated with the simple point charge water (SPC) [42] and neutralised with NaCl in a concentration of 150 mM. The energy minimisation was performed with the steepest descent. The first step of equilibration was performed in an NVT ensemble for 100 ps. The system was coupled to the V-rescale thermostat [43] at 298 K. All bonds were constrained with the LINCS algorithm [44], the cut-off for short-range non-bonded interactions was set at 1.4 nm, and long-range electrostatics were calculated using the particle-mesh Ewald (PME) algorithm [45]. The second step of equilibration was performed in the NPT ensemble coupled to Parrinello–Rahman barostat [46] to isotropically regulate the pressure for 100 ps. The pulling of piperine from the IL6 pocket was performed without restraints to allow the protein conformational changes. The reaction coordinate ξ was chosen as being the distance between Phe171 carbon atom (CA index 1535) and piperine carbon atom (CAL 1716) (Figure S3). Piperine was pulled away from the IL6 binding site in Z direction until the reaction coordinate reached 7 nm (Figure S2a), using a spring constant of 800 kJ/mol^−1^ nm^−2^ and a pull rate of 0.01 nm/ns. The samplings of the pullings were analysed to ensure a good sampling (Figure S2b). The potential of mean force (PMF) profile [47] along the reaction coordinate was calculated with WHAM method [48]. Statistical errors were estimated with bootstrap analysis, with 1000 bootstraps properly autocorrelated.

The stability of the protein and the complex was analysed by RMSD and radius of gyration profiles obtained from an average of three independent simulations of 800 ns duration.

## 4. Conclusions

The present work investigated the mode of binding of the IL6-piperine complex by means of experimental and computational molecular biophysical methodologies. Steady-state fluorescence spectroscopy revealed the complex formed via a static quenching mechanism. Moreover, the spectroscopic results along with van’t Hoff analyses revealed a spontaneous complexation (ΔG < 0 kJ/mol) driven by non-specific interaction with a complex affinity in the order of 10^4^ M^−1^. Molecular docking and dynamics confirmed the binding site through the prediction of ΔG by umbrella sampling along with the WHAM method. The stability of the complex was verified in molecular dynamics during 800 ns of simulation performing RMSD and hydrodynamic radius calculations. Furthermore, molecular modelling reinforced the findings obtained experimentally regarding the non-specific interactions that drove the complexation, showing that during the 800 ns of molecular dynamics, the complex formed either one or zero hydrogen bonds. Although piperine has three hydrogen bond acceptors, only a single hydrogen bond formed. Further chemical modifications could be made to the piperine structure in order to add hydrogen bond donors and consequently increase the affinity for the IL6 binding site. Circular dichroism aided by molecular dynamics revealed IL6 experienced a reduction in α-*helices*, pointing out that this reduction may cause changes in IL6 activity. In conclusion, a multispectroscopic evaluation aided by molecular docking and dynamic elucidated in detail the mode of binding of piperine to IL6, which may further support small-molecule drug discovery teams in the early stages of drug development research.

## Figures and Tables

**Table 1:** Stern–Volmer constant (K_SV_) and binding constant (K_a_) for the IL6-piperine complex at 288, 298, and 308 K.

Temperature (K)	Stern–Volmer (K_SV_) × 10^4^ M^−1^	Binding (K_a_) × 10^4^ M^−1^
288	3.69 ± 0.09	4.2 ± 0.3
298	3.67 ± 0.08	4.3 ± 0.5
308	3.68 ± 0.07	4.3 ± 0.5

**Table 2:** Thermodynamic parameters of the complex IL6-piperine at the temperatures of 288 K, 298 K and 308 K.

T (K)	ΔG (kJ/mol)	ΔH (kJ/mol)	T.ΔS (kJ/mol)
288	−14 ± 3	2.5 ± 0.2	16 ± 3
298	−14 ± 3	2.5 ± 0.2	17 ± 3
308	−15 ± 3	2.5 ± 0.2	17 ± 3

## Supplementary Materials

The following supporting information can be downloaded at: https://www.mdpi.com/article/10.3390/ijms23147994/s1.
