# Supplementary material for: A Computational–Experimental Investigation of the Molecular Mechanism of Interleukin-6-Piperine Interaction
Source: Int J Mol Sci. 2022 Jul 20;23(14):7994. doi: 10.3390/ijms23147994 (PMC9323498; doi:10.3390/ijms23147994)
Supplement: Supplementary file 1 [file ijms-23-07994-s001.zip › ijms-1814384-supplementary.pdf]

International Journal of *Molecular Sciences*

MDPI

Supplementary material

A Computational–Experimental Investigation of the Molecular Mechanism of Interleukin-6−Piperine Interaction

Ana Paula Ribeiro Povinelli [1], Gabriel Zazeri [1,*], Alan M. Jones [2] and Marinônio Lopes Cornélio [3,*]

[1] Federal Institute of Education, Science and Technology of Mato Grosso, Campo Novo do Parecis, 78360-000, Brazil; ana.povinelli@colaborador.ifmt.edu.br
[2] School of Pharmacy, Institute of Clinical Sciences, College of Medical and Dental Sciences, University of Birmingham, Edgbaston, Birmingham B15 2TT, UK; a.m.jones.2@bham.ac.uk
[3] Departamento de Física, Instituto de Biociências, Letras e Ciências Exatas (IBILCE), UNESP, Rua Cristovão Colombo 2265, São José do Rio Preto 15054-000, Brazil
* Correspondence: gabriel.zazeri@colaborador.ifmt.edu.br (G.Z.) ; m.cornelio@unesp.br (M.L.C.)

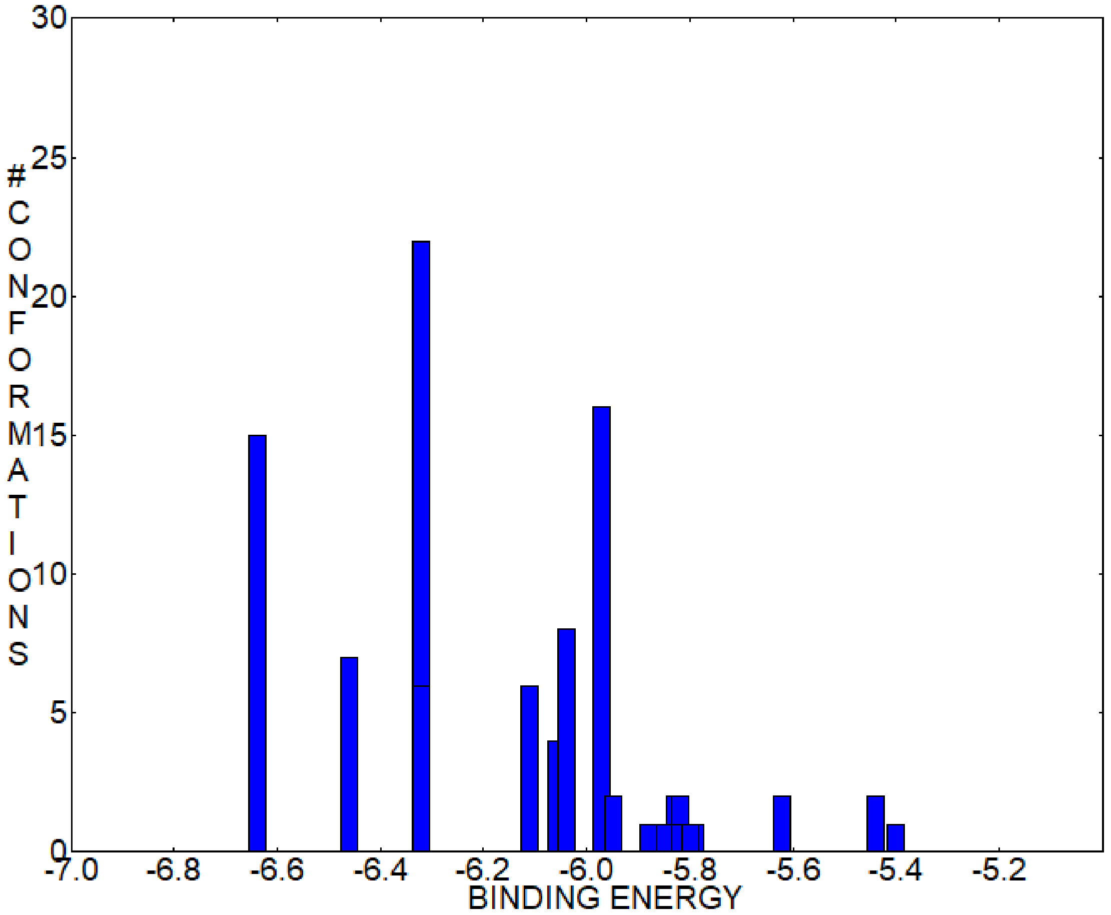

Figure S1: Molecular docking clusters with their respective energy scores

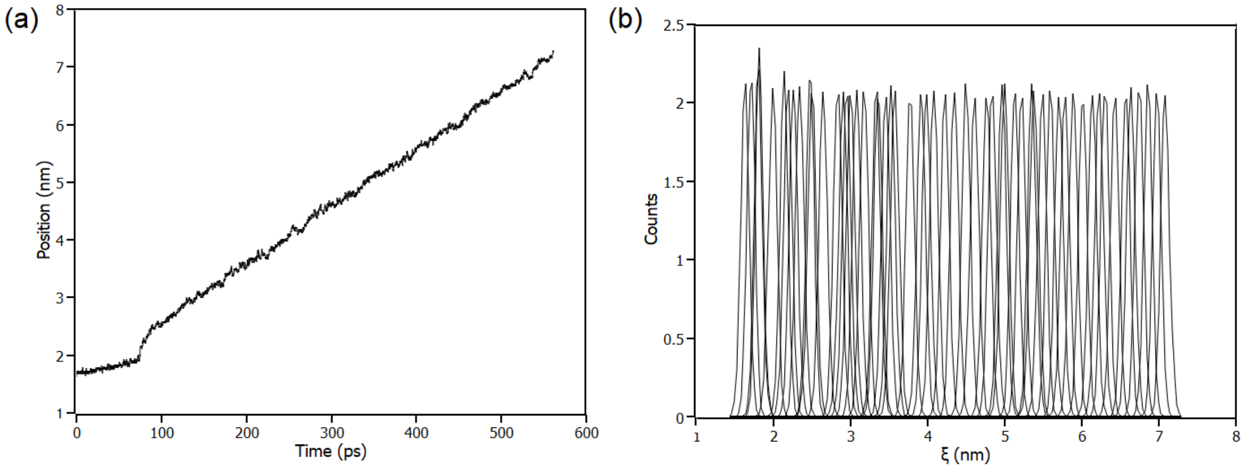

Figure S2: (a) Pulling profile during the pulling simulation. Y-axis is the value of reaction coordinate (ξ) and x-axis is the time of simulation. **(b)** Configuration histograms of the pulling in z-axis with the windows distance = 0.1 nm.

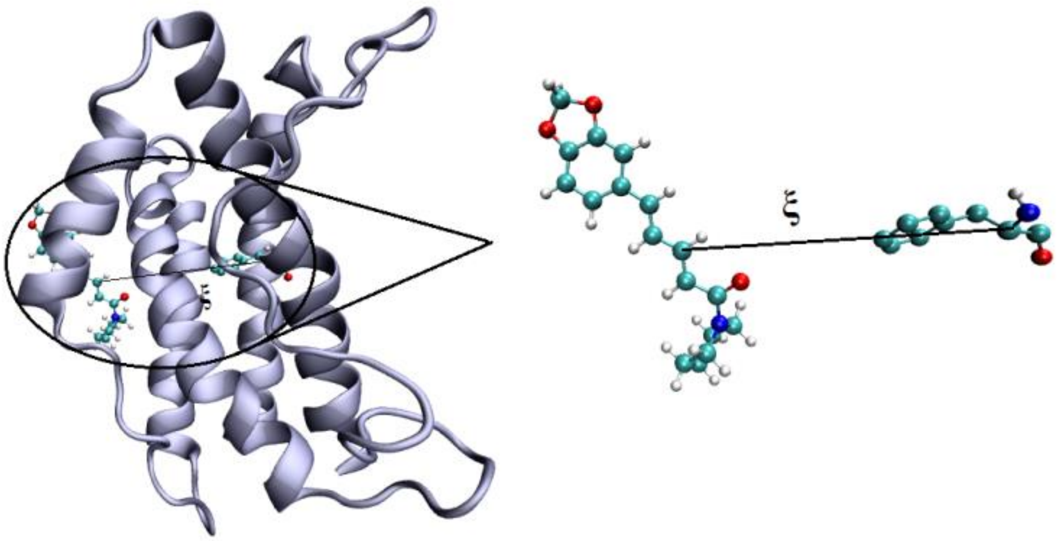

Figure S3: The atoms picked to define the reaction coordinate (ξ) in umbrella sampling calculations. Zoomed in view of piperine and Phe171.

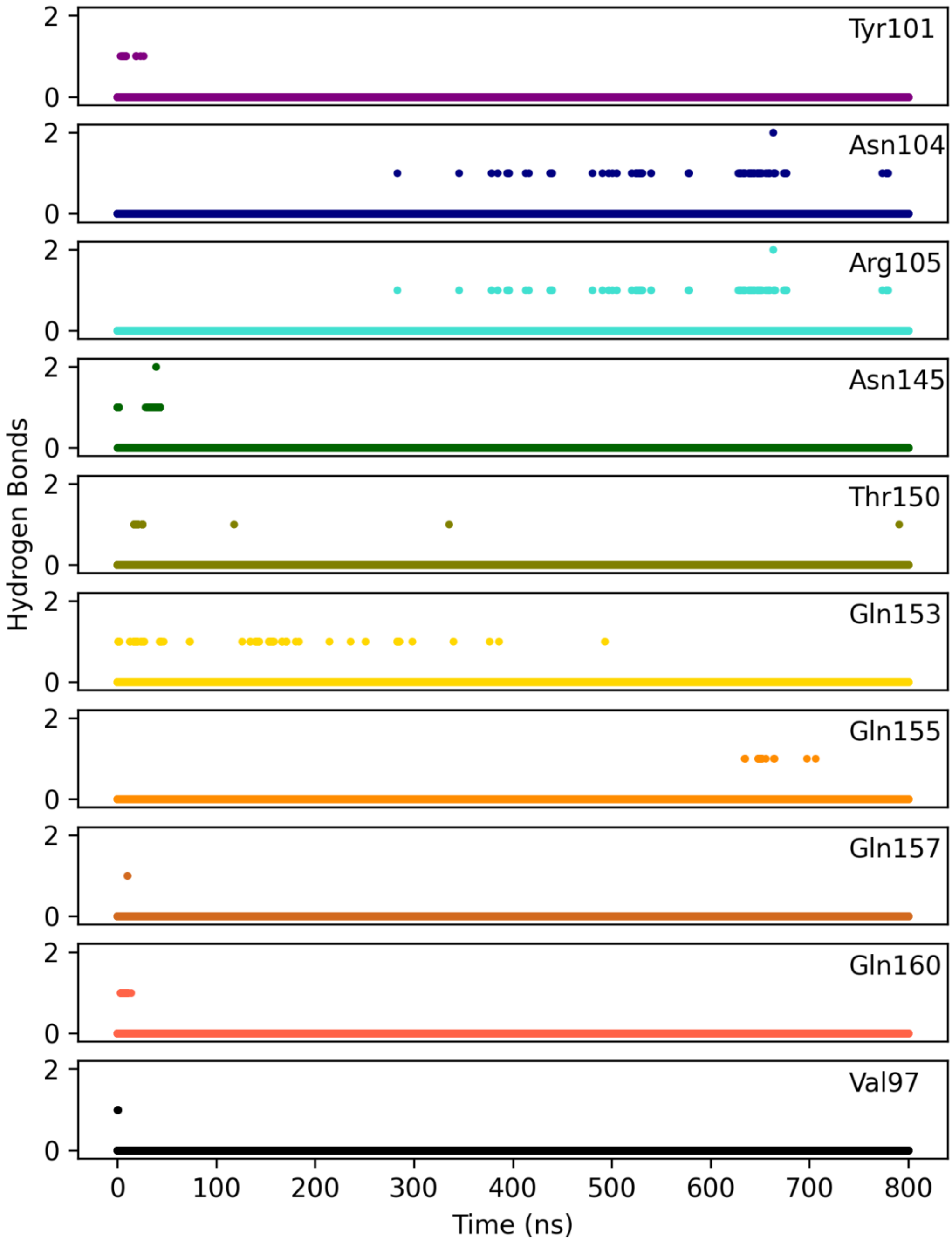

Figure S4: Hydrogen bonds performed during 800 ns of simulation.

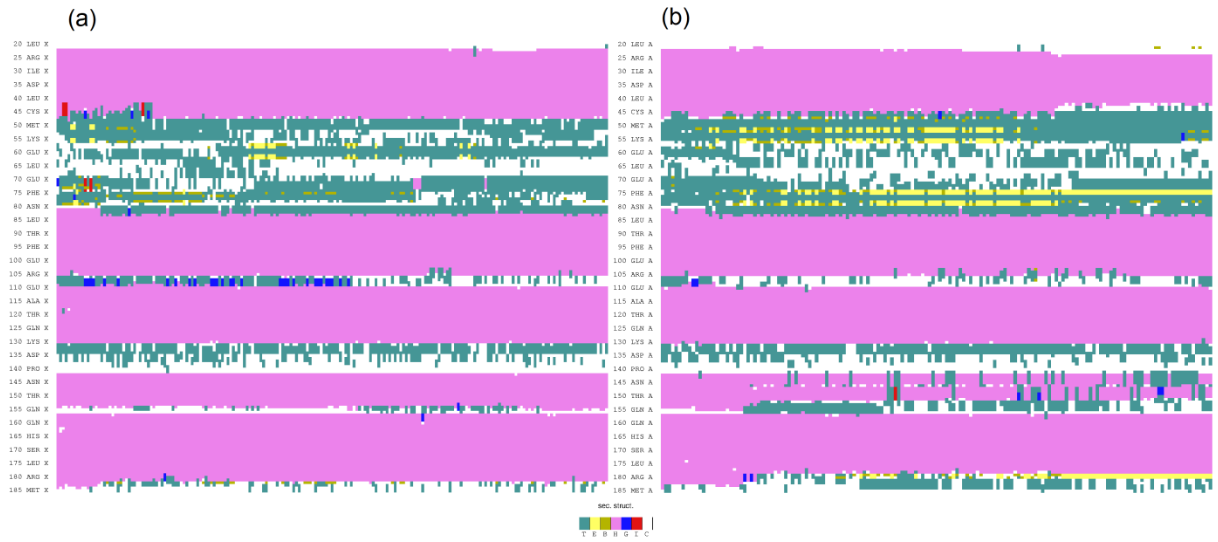

Figure S5: Global secondary structures of IL6 calculated during the molecular dynamics simulations in **(a)** the absence of piperine and **(b)** piperine inside the binding pocket.
